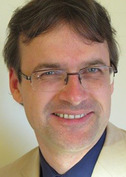# Extending Designed Linear Biocatalytic Cascades for Organic Synthesis

**DOI:** 10.1002/cctc.201801063

**Published:** 2018-08-28

**Authors:** Somayyeh Gandomkar, Anna Żądło‐Dobrowolska, Wolfgang Kroutil

**Affiliations:** ^1^ Institute of Chemistry University of Graz Heinrichstrasse 28 Graz 8010 Austria

**Keywords:** Cascade, biocatalyst, biotransformation, linear biocatalytic cascades, multi-step reaction

## Abstract

Artificial cascade reactions involving biocatalysts have demonstrated a tremendous potential during the recent years. This review just focuses on selected examples of the last year and putting them into context to a previously published suggestion for classification. Subdividing the cascades according to the number of catalysts in the linear sequence, and classifying whether the steps are performed simultaneous or in a sequential fashion as well as whether the reaction sequence is performed *in vitro* or *in vivo* allows to organise the concepts. The last year showed, that combinations of *in vivo* as well as *in vitro* are possible. Incompatible reaction steps may be run in a sequential fashion or by compartmentalisation of the incompatible steps either by using special reactors (membrane), polymersomes or flow techniques.

## Graphical abstract

**Chasing waterfalls**! Artificial cascades involving biocatalysts are selected from the last year and are classified. The recent examples showed, that even combinations of *in vivo* as well as *in vitro* cascades are possible. Incompatible reaction steps may be run in a sequential fashion or by compartmentalisation of the incompatible steps.

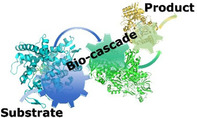

## 1 Introduction

Biocatalytic cascades^1^ have been recognized as a very useful tool to circumvent possible limitations of single step transformations (non‐favored equilibrium, inhibition, unstable substrate/intermediate) or make a multi‐step synthesis more efficient by avoiding the isolation of intermediates, thereby reducing reaction time, saving resources (solvents) and minimizing waste. As a result of the high impact of cascade, many papers have been published within the last year including various reviews with different foci,1a,^2^ for instance addressing strategies and perspectives for assembling multi‐enzyme systems,2b,2c *in vitro* and *in vivo* approaches to *de novo* multi‐enzyme pathways,2d or challenges and opportunities of cascading enzymatic microreactors.2e

The various reviews as well as new designed or/and improved cascades reveal beside the many advantages of cascade also the challenges, which refer for instance to the compatibility of the individual reactions steps within a sequence. Obviously, the simultaneous combination of several steps in one pot requires that all enzymes and reagents do not lead to any cross reactivity. This is an important requirement additionally to the need that the reactions conditions such as pH and temperature are suitable for all enzymes involved. If this is not the case, the sequence may be performed in a stepwise/sequential fashion, thus the reagents of a later step are added when the preceding steps are finished. Alternatively, a flow system may allow to provide something comparable to compartmentalization. Consequently, it is of special interest how compartmentalization is realized in nature.^3^ We will discuss in this review in the chapters about sequential cascades and flow system which challenges had to be circumvented to achieve finally a successful cascade. It is worth to mention that beside cascades in flow (see section 3.3) also for simultaneous *in vitro* cascades compartmentalization has been realized (see section 3.1.3, Scheme 23).

Following the classification of cascades suggested recently,1a examples were selected from the last year not reviewed before to demonstrate and evaluate this classification. It became clear, that novel concepts have been developed which have not been considered before like the combination of *in vivo* and *in vitro* systems (see section 5). For *in vivo* cascades the cofactor recycling is performed by the metabolism of the living organism. Furthermore, since it was suggested to classify the sequences accordingly to the number of enzymes in the linear sequence, it turned out that this description might not be sufficient in case of fusion proteins able to perform several different reactions steps. Therefore, it might be worth to consider both the number of enzymes and the number of activities they can perform. Consequently, the review is structured following the proposed classification: first, according to the number of enzymes involved in the linear sequence; next, whether the subsequent reactions are spontaneous or need a catalyst. Here we just consider cascades involving a biocatalyst. Furthermore, the cascades are grouped depending whether the steps of the sequence are run simultaneously or the reaction has to be divided into stages performed sequentially. Importantly, it was also distinguished between cascades performed *in vitro* and *in vivo*.

## 2 Spontaneous Cascades Triggered by a Single Enzyme

**Scheme 1:**
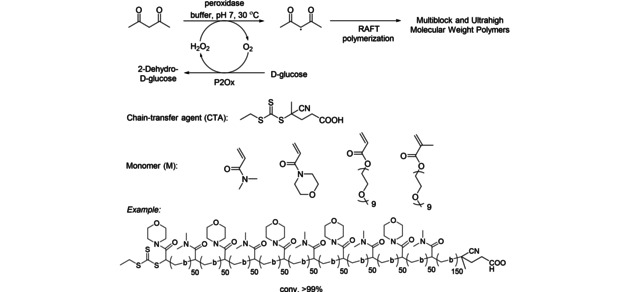
Horseradish peroxidase‐triggered cascade for the synthesis of multiblock polymers in open vessels, and ultrahigh molecular weight polymers (UHMW) in closed vessels (RAFT polymerization: reversible addition‐fragmentation chain‐transfer).

Artificial cascades involving a single enzyme in the linear reaction sequence make up only a small fraction of all cascades. Either a single enzyme can catalyze several steps (see section 3.1.1) or it has only a single activity and initiates a domino reaction, thus a spontaneous subsequent reaction (sequence). Examples for the latter case are given here. For instance, a peroxidase catalyzed the formation of radicals which polymerized leading to well‐defined multiblock polymers in open vessels and Ultrahigh Molecular Weight Polymers (UMWP) in closed vessels (Scheme 1).^4^ The required hydrogen peroxide for the peroxidase was produced in a parallel reaction *via* oxidation of glucose by the oxidase P2Ox prior addition of the horseradish peroxidase (HRP). The latter catalyzed then the formation of acetyl acetone (ACAC) radicals, which spontaneously underwent reversible addition‐fragmentation chain‐transfer (RAFT) polymerization. The cascade allowed the efficient synthesis of sequence‐controlled multiblock (up to 10 blocks) copolymers in open vessels and well‐defined UHMW polymers with molecular weights up to 2.3×10^6^  g/mol.

**Scheme 2:**
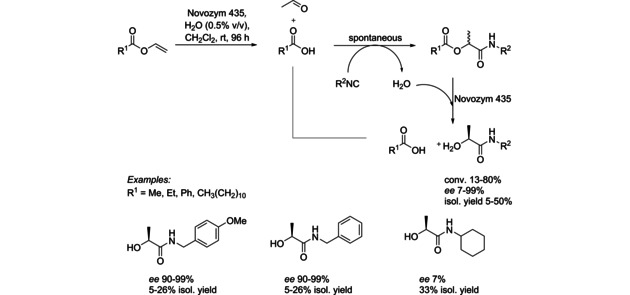
Putrescine transaminase‐triggered domino reaction *via* cyclization and reaction with phenyl acetaldehyde.

In another example putrescine transaminase was employed to deaminate terminal aliphatic diamines (Scheme 2).^5^ The diamines were thereby converted into reactive amino aldehydes, which spontaneously cyclized to the corresponding cyclic imines driving the reaction towards product formation. Pyruvate and α‐ketoglutarate were employed as amine acceptors. The formed imine reacted further with 1‐(*p*‐methoxyphenyl)‐2‐propanone leading to 2,3‐dihydro‐1H‐indolizinium motifs, notably the antibacterial and antifungal alkaloid ficuseptine (isol. yield 50 %) and its analogue (isol. yield 40 %).

**Scheme 3:**
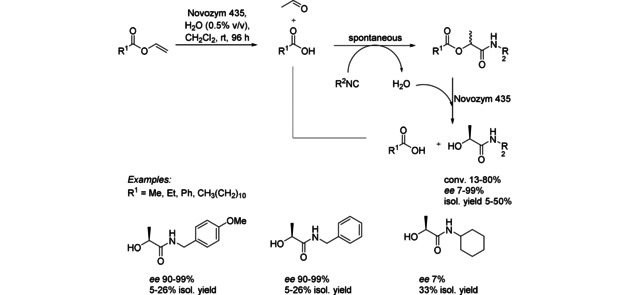
Lipase‐triggered cascade in combination with a Passerini reaction.

Although the next cascade is triggered by an enzyme with a single activity (hydrolysis), the enzymes is involved in a third step performing another hydrolytic step leading to α‐hydroxy carboxamide (Scheme 3). In a first hydrolysis step the lipase generated carboxylic acid and vinyl alcohol, which spontaneously tautomerized to acetaldehyde.^6^ The second step represents a multi‐component reaction (Passerini reaction) of acetaldehyde, carboxylic acid and isocyanide. Finally, an enzymatic kinetic resolution catalyzed by the same lipase occurred. The cascade was performed on a 25 mM scale in organic solvents, as well as in water leading to products with 5–50 % isolated yields and *ee* up to >99 %. Combination of diversity delivered by multicomponent reactions with the selectivity of enzymes is a powerful concept providing complex compounds with high enantiomeric purity.^7^

## 3 *In Vitro* Cascades Requiring for Each Step a Biocatalyst

### 3.1 Simultaneous Mode

#### 3.1.1 Single Catalyst with Various Activities

Nature uses proteins with multiple activities (e. g. polyketide synthase) for multi‐step reactions to facilitate more rapidly the conversion of substrates through intermediates to the final product with less side‐product formation. In a similar fashion, the fusion of biocatalysts may be advantageous for enzyme cascade reactions. The first two cascades mentioned here have been reported previously using individual enzymes, therefore the cascades were discussed previously as cascades involving two or three enzymes each with a single activity, while here a single protein displays two or three different activities.

**Scheme 4:**
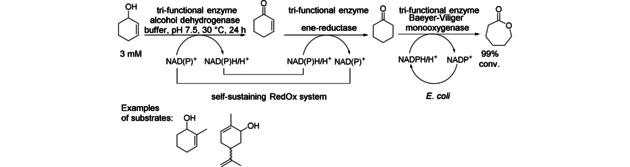
*In vivo* cascade for conversion of cyclic allylic alcohols to their corresponding lactones by using a fused alcohol dehydrogenase, an enoate reductase and a Baeyer‐Villiger monooxygenase (CHMO).

A fusion protein consisting of two enzyme activities, namely an enoate reductase (ERED) and a Baeyer‐Villiger monooxygenase (BVMO) combined with non‐fused alcohol dehydrogenase (ADH), enabled the synthesis of (chiral) lactones starting from allylic alcohols as substrates (Scheme 4).^8^ The domain order and various linkers studies have been done and the best results were obtained for the ERED xenobiotic reductase B from *Pseudomonas putid*a and the cyclohexanone monooxygenase (CHMO) from *Acinetobacter* sp. In addition, the performance of the fusion enzymes was improved by using a double mutant of the CHMO, C376L/M400I. Formation of 2 mM of dihydrocarvone lactone after 3 h with an *E. coli* expressing the CHMO‐QC G XenB fusion protein and *LK*‐ADH in whole cell was observed, while by using the three enzymes without fusion, 1.2 mM product was formed. Some research was also done on the kinetic model for the simulation and optimization of such cascades.^9^ In order to simulate the change in concentration of intermediates and products during the *in vivo* cascade reactions, an adapted Michaelis‐Menten parameter from *in vitro* experiments with isolated enzymes was estimated and used. Remarkably, the model indicated that the fastest enzyme is rate determining due to the unexpectedly low concentration of the active form, opening up reversible reaction channels towards side products. Other experimental evidence proved that the performance of the *in vivo* cascade was drastically throttled at low intracellular concentration of flavin and nicotinamide cofactors.

**Scheme 5:**
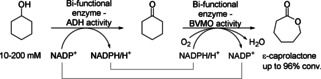
Conversion of cyclohexanol to *ϵ*‐caprolactone using a bifunctional protein [alcohol dehydrogenase (ADH) and a Baeyer‐Villiger monooxygenase (BVMO)].

ϵ‐Caprolactone is a valuable chemical for polymer synthesis that is being used for the production of biodegradable polymers, such as polycaprolactone, and as a precursor to ϵ‐caprolactam. Beside the approach mentioned above a redox self‐sufficient bifunctional enzyme possessing alcohol dehydrogenase and Baeyer‐Villiger monooxygenase activity was designed (Scheme 5).^10^ Molecular oxygen was the only stoichiometric co‐substrate in the overall process. By using *Tb*ADH‐*Tm*CHMO fusion (*Tm*CHMO from *Thermocrispum municipale* and *Tb*ADH from *Thermoanaerobacter brockii*), >99 % conversion of 200 mM cyclohexanol to *ϵ*‐caprolactone was achieved in 24 h, with >13,000 turnovers per fusion enzyme molecule. Transformations with high substrate concentrations showed difficulties due to solubility issues, limited enzyme stability, substrate and product inhibition. Consequently, substrate and product inhibition was circumvented by substrate feeding with a syringe pump at 5 μL/h and CAL‐A lipase was added to convert the product *ϵ*‐caprolactone to oligo‐caprolactone, thereby reducing product inhibition. For 500 mM of substrate, 30–40 % of conversion was observed by using 20 to 40 μM of fusion enzymes after 48 h.

This cascade has been also optimized further by improved expression of the individual enzymes^11^ or improved reaction engineering^12^ which belongs to the chapter dealing with two individual enzymes (each with a single activity).

**Scheme 6:**
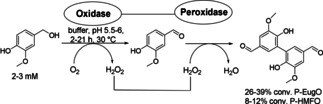
Conversion of vanillyl alcohol to divanillin through an oxidative cascade by using a fused oxidase‐peroxidases (P‐oxidases).

Another example of bifunctional proteins encompasses a fused oxidase and a peroxidase. A set of bifunctional oxidase–peroxidases has been prepared by fusing a bacterial peroxidase (*Svi*DyP from *Saccharomonospora viridis* DSM 43017, EC 1.11.1.19) with an oxidase [e. g. eugenol oxidase from *Rhodococcus* sp. strain RHA1 (EugO) and 5‐hydroxymethylfurfural oxidase from *Methylovorus* sp. strain MP688 (HMFO)]. The peroxidase fusion with EugO and HMFO was used for dioxygen‐driven, one‐pot, two‐step cascades to convert vanillyl alcohol into divanillin (Scheme 6) and eugenol into lignin oligomers, respectively.^13^

#### 3.1.2 Two Catalysts in the Linear Sequence

##### 3.1.2.1 Redox Cascades – 2 Redox Steps

###### 3.1.2.1.1 Hydrogen‐borrowing Cascades

Hydrogen‐borrowing cascades are typically made up by two steps, whereby one option is that an oxidation and a reduction step are combined involving two enzymes and a cofactor which interconnects the two redox steps; thus, the oxidation and reduction steps are linked to each other in general *via* the exchange of a hydride, which is abstracted in the oxidation step and consumed in the reduction step. The interconnection *via* the cofactor of two redox steps can also be realized in a second option, combining a hydroxylation (consuming the reduced nicotinamide cofactor) and an alcohol oxidation (producing the reduced cofactor). These systems are sensitive to side reactions where the redox cofactor [e. g., NAD(P)H] gets consumed by other enzymes present.

**Scheme 7:**
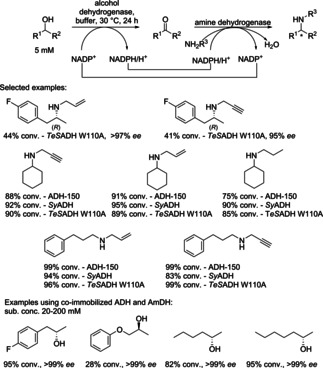
Functional group interconversion of alcohols to secondary amines (or alkylation of a primary amine) *via* a hydrogen‐borrowing cascade using alcohol and amine dehydrogenases.

In a recent example, the reductive aminase from *Aspergillus oryzae* (*Asp*RedAm; a non‐stereoselective NADPH‐dependent amine dehydrogenase) was combined with an alcohol dehydrogenase [either metagenomic ADH‐150, an ADH from *Sphingobium yanoikuyae* (*Sy*ADH), or a variant of the ADH from *Thermoanaerobacter ethanolicus* (*TeS*ADH W110A)] in a redox‐neutral cascade for the formal alkylation of amines using primary and secondary alcohols (Scheme 7). Aliphatic and aromatic secondary amines were obtained in up to 99 % conversion, as well as chiral amines directly from the racemic alcohol in up to >97 % *ee*, releasing water as the only byproduct.^14^

The efficiency of the hydrogen borrowing biocatalytic amination was enhanced by co‐immobilizing of an alcohol dehydrogenase from *Aromatoleum aromaticum* (*AA*‐ADH) and a chimeric amine dehydrogenase (Ch1‐AmDH) on controlled porosity glass Fe^III^ ion‐affinity beads (EziG Fe‐amber). EziG Fe^III^ ion affinity beads were used to bind selectively His‐tagged enzymes from crude enzyme cell extracts as well. The recyclability (5 cycles) of the dual‐enzyme system was demonstrated with total turnover numbers of >4000 and >1000 for ADH and AmDH, respectively, which represented an improvement of 2 to 15‐fold compared to previous studies with free enzymes in solution. A set of (*S*)‐configured alcohol substrates was aminated with up to 95 % conversion and >99 % *ee* (*R*) (Scheme 7, bottom). Preparative‐scale amination of (*S*)‐phenylpropan‐2‐ol resulted in 90 % conversion and 80 % yield of the product in 24 h.^15^ The cascade was extended by starting with a hydroxylation reaction prior amination (see section 3.1.3, Scheme 21).^16^

**Scheme 8:**
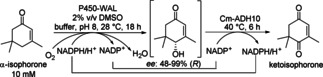
Single‐cell double‐oxidation cascade of *α*‐isophorone to ketoisophorone using *E. coli* co‐expressing P450‐WAL and Cm‐ADH10.

An example for a cascade with interconnected two oxidation steps is provided below. The double oxidation of *α*‐isophorone (*α*‐IP) to ketoisophorone was enabled by combining a P450 monooxygenase and an ADH (Scheme 8).^17^ Variants of the self‐sufficient P450cam‐RhFRed with improved activity have been identified to perform the regio‐ and enantioselective allylic oxidation of *α*‐IP to 4‐hydroxy‐*α*‐isophorone as the first step. For the second step *Cm*‐ADH10 from *Candida magnolia* was identified to be most effective. The synthesis of ketoisophorone was demonstrated both as a one‐pot sequential process and as a simultaneous process employing designed cells co‐expressing the two biocatalysts, with a productivity of up to 1.4 g/L.d. Higher TTNs were reached by a simultaneous process compared to the stepwise process (3421 vs. 1567), due to a more effective cofactor recycling system and/or a positive effect resulting from the removal of the intermediate. The simultaneous two‐step double allylic oxidation of α‐IP, was successfully scaled up (100 mg *α*‐IP, 65 mL total volume) and after 24 h, 80 % conversion for the substrate, 87 % recovery and a total isolated yield of 56 % were obtained.

###### 3.1.2.1.2 Oxidation Followed by Reduction

**Scheme 9:**
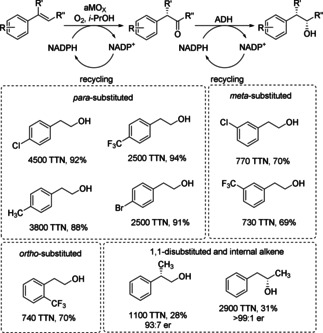
aMOx (anti‐Markovnikov oxidase) in combination with an alcohol dehydrogenase (ADH) to convert alkenes to their corresponding anti‐Markovnikov alcohols in a simultaneous two step cascade.

Anti‐Markovnikov oxidation of alkenes is a long‐standing challenge in chemistry and identifying suitable biocatalytic methods could simplify synthetic routes. Recently, an engineered cytochrome P450 enzyme from the rhodobacterium *Labrenzia aggregata* (P450LA1) was developed by directed evolution, which catalyzed an anti‐Markovnikov oxidation of styrenes to the corresponding carbonyl compound (Scheme 9).^18^ The final best performing variant displayed eight amino acid exchanges (A103L, M118L, R120H, V123I, I326V, V327M, H385V, and M391L), overall substituting 3 % of the heme domain amino acids. Dioxygen was required as the terminal oxidant and selectivity for anti‐Markovnikov oxidation was achieved over the kinetically favored alkene epoxidation by trapping high‐energy intermediates and catalyzing an oxo transfer, including an enantioselective 1,2‐hydride migration. In order to access a variety of anti‐Markovnikov functionalization reactions, the oxidation can be combined with other catalysts in synthetic metabolic pathways, like with an alcohol dehydrogenase leading overall to formal hydration of the alkene (Scheme 9).

A related approach for the hydration of alkenes required three enzymes/three steps and went through epoxidation, isomerization and reduction (Scheme 35).

###### 3.1.2.1.3 Two oxidation steps

**Scheme 10:**
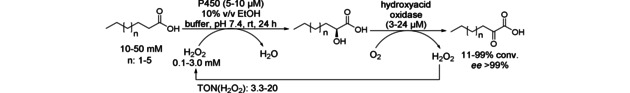
Enzymatic oxidation of fatty acids to *α*‐ketoacids *via* internal H_2_O_2_ recycling in a simultaneous two‐step cascade by using P450 monooxygenase in peroxygenase mode and *α*‐hydroxyacid oxidase.

The functionalization of bio‐based chemicals is essential to allow valorization of natural carbon sources. An atom‐efficient biocatalytic oxidative cascade was developed for the conversion of saturated fatty acids to *α*‐ketoacids (Scheme 10).^19^ A P450 monooxygenase [(P450 from *Clostridium acetobutylicum* (P450_CLA_) or P450 from *Sphingomonas paucimobilis* (P450_SPα_)] was employed for regioselective α‐hydroxylation of fatty acids exploiting the peroxide shunt. This oxyfunctionalization step was combined with enantioselective oxidation by α‐hydroxyacid oxidase(s) [(*S*)‐specific α‐hydroxyacid oxidase from *Aerococcus viridans* (*α*‐HAO) and D‐lactate oxidase from *Gluconobacter oxydans* (GO‐LOX)] enabling the internal recycling of the oxidant H_2_O_2_ required for the first step from molecular oxygen produced in the second step. Furthermore, removal of H_2_O_2_ minimized degradation of the labile ketoacid product.

Employing P450_CLA_ and (*S*)‐α‐HAO with substoichiometric amounts of H_2_O_2_ revealed that the conversion to the oxo product is limited by non‐matching stereoselectivity of the hydroxylation and alcohol oxidation step. Since P450_CLA_ showed low stereoselectivity (max. 36 % *ee* towards (*S*)‐hydroxy acid), two stereocomplementary oxidases [(*S*)‐α‐HAO and GO‐LOX] were used in the second step of the cascade to reach then 98 % of conversion by using 0.3 equivalent of H_2_O_2_ and octanoic acid (10 mM). By using 0.1 equivalent of H_2_O_2_, 77 % of conversion with TON of H_2_O_2_ 7.7 was observed, therefore it was concluded that the recycling was more efficient at low peroxide concentration. The stereoselective P450_SPα_ transformed octanoic acid to the corresponding (*S*)‐α‐hydroxy acid in optical pure form (>99 % *ee*) avoiding the need for two stereocomplementary oxidases. Scale‐up allowed isolation of final product in 91 % yield and the cascade was applied to fatty acids of various chain lengths (C6:0 to C10:0).

##### 3.1.2.2 Redox Cascades – One Redox Step

###### 3.1.2.2.1 Reductive Amination Followed by Acylation

**Scheme 11:**
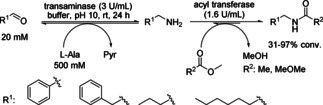
Transamination followed by amine acylation. The transaminase from *Silicibacter pomeroyi* (*Sp*‐ATA) and the acyl transferase from *Mycobacterium smegmatis* (*Ms*AcT) enabled the transformation of benzaldehyde into *N*‐benzylacetamide using either methyl acetate or methyl methoxyacetate as acyl donor.

Amides were prepared from the corresponding aldehydes and ketones in aqueous solution by using the transaminase from *Silicibacter pomeroyi* (*Sp*‐ATA) in combination with acyl transferase from *Mycobacterium smegmatis* (*Ms*AcT), which is able to perform trans‐acylations in aqueous solution (Scheme 11). L‐alanine was used as amine donor for the transaminase in the first step. Only an amino acid as amine donor is compatible with the *Ms*AcT catalyzed amide formation, since other amine donors might get acylated. Furthermore, due to high solubility of L‐alanine in water, it can be used in excess to displace the equilibrium towards product formation. A preparative synthesis of *N*‐benzyl‐2‐methoxyacetamide led to formation of 134 mg of *N*‐benzyl‐2‐methoxyacetamide from benzaldehyde with 92 % conversion and 75 % isolated yield.^20^

###### 3.1.2.2.2 Oxidation followed by C−C Bond or Amide Formation

**Scheme 12:**
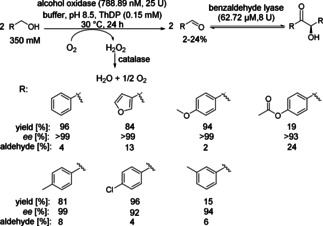
Alcohol oxidation followed by C−C bond formation involving the alcohol oxidase from *Pichia pastoris* (*Pp*AOX) and benzaldehyde lyase from *Pseudomonas fluorescens* (*Pf*BAL).

Chiral α‐hydroxy ketones are valuable building blocks especially in fine chemistry and pharmaceutical applications. Biocatalytic cascades for the synthesis of chiral α‐hydroxy ketones starting from aliphatic/benzylic alcohols oxidized to their corresponding aldehydes followed by subsequent carboligation have been exemplarily evaluated (Scheme 12). This approach avoids the handling and addition of the reactive aldehyde enabling the addition of high alcohol substrate concentrations in one liquid phase while maintaining enzyme activity.

The cascade consisting of alcohol oxidase from *Pichia pastoris* (*Pp*AOX) and benzaldehyde lyase from *Pseudomonas fluorescens* (*Pf*BAL) was reported for the stereoselective synthesis of (*R*)‐benzoins.^21^ Benzylalcohol and *p*‐substituted derivatives were converted to the enantiomerically pure benzoins in good to excellent yields (81–96 %), while other substitution patterns led to significantly reduced conversions and optical purities of the final products. Highest conversions were obtained by the molar ratio of 1 : 2 of *Pp*AOX/*Pf*BAL. In all cases tested, the final product was easily separated from the reaction mixture by filtration. A semi‐preparative reaction was performed with up to 500 mM benzyl alcohol (54 g/L), which was added in ten 50 mM portions, due to the limited solubility and possible substrate inhibition on *Pp*AOX. The raw product contained 5.4 mM of the intermediate aldehyde, which was removed by evaporation under reduced pressure and overall 4.6 g (83 % yield) of enantiomerically pure (*R*)‐ benzoin was obtained.

**Scheme 13:**
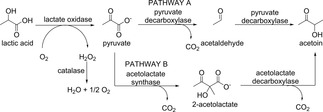
Three‐step enzymatic cascades for the efficient conversion of racemic lactate into acetoin.

Another cascade involving alcohol oxidation and C−C bond formation leading to α‐hydroxy ketones was reported starting from lactate (Scheme 13). The combination of L‐lactate oxidase (L‐LOX) from *Pediococcus* sp. and D‐Lactate oxidase (D‐LOX) from *Gluconobacter oxydans* 621H oxidized racemic lactate to pyruvate (Pathway A, Scheme 13). In the second step pyruvate decarboxylase (PDC) from *Zymomonas mobilis* was applied to produce acetoin from pyruvate. Finally, 9.8 mM acetoin was produced from 21.1 mM racemic lactate at a yield of 92.7 %.^22^

Many other biotransformations have also been reported for the production of acetoin from lactate.^23^ For example, enzymes immobilized in a Ca‐alginate gel were designed for the removal of lactic acid from grass silage press juice. LOX was utilized in the first step to oxidize lactic acid to pyruvate, followed by acetolactate synthase (ALS) to produce 2‐acetolactate, which was then decarboxylated by acetolactate decarboxylase (ALDC) to form acetoin (Pathway B, Scheme 13). The reaction was performed with a continuous, pH‐dependent substrate feed under oxygenation. With 50 mM lactic acid solution, a yield of 91 % was obtained over 6 h reaction time. By using grass silage press juice as the titrant, which was diluted to achieve an equivalent amount of lactic acid, yields of 49 % were obtained within the same reaction time.^23^

In addition, two other cascades involving two steps for acetoin production were demonstrated on analytical scale giving acetoin with 34 % yield,^24^ or starting from ethanol, 44 mM of acetoin was obtained with 89 % of the theoretical yield.^25^

**Scheme 14:**
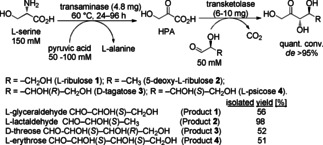
Simultaneous two‐step synthesis of naturally rare L‐erythro (3*S*,4*S*) ketoses catalyzed by coupling a thermostable L‐α‐transaminase from the thermophilic bacterium *Thermosinus carboxydivoran* and a transketolase from *Geobacillus stearothermophilus*.

Naturally rare L‐erythro (3*S*,4*S*)‐ketoses were synthesized by a simultaneous two enzyme cascade using a thermostable L‐α‐transaminase from the thermophilic bacterium *Thermosinus carboxydivoran* (TA_tca_) and a transketolase from *Geobacillus stearothermophilus* (TK_gst_) at elevated temperature (60 °C) (Scheme 14).^26^ L‐ribulose, 5‐deoxy‐L‐ribulose, D‐tagatose and L‐psicose were obtained on a preparative scale with excellent stereoselectivities and good yields. The final products were obtained through the stereospecific C−C bond formation catalyzed by TK_gst_ from (2*S*)‐hydroxylated aldehydes and *β*‐hydroxypyruvate (HPA), whereby the latter is generated *in situ* from L‐serine by the transaminase employing pyruvate as amine acceptor.

**Scheme 15:**
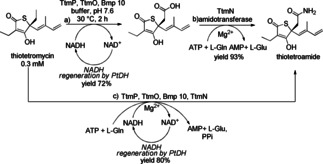
Biosynthetic conversion of thiotetromycin to thiotetroamide C by the oxidation–amidation cascade catalyzed by the TtmP P450 and the TtmN amidotransferase. Abbreviations: AMP=adenosine monophosphate; ATP=adenosine triphosphate; bmp 10: ferredoxin reductase; NADH/NAD^+^=reduced/oxidized nicotinamide adenine dinucleotide; PPi=inorganic pyrophosphate; PtDH=phosphite dehydrogenase; TtmO: electron shuttle ferredoxin.

Although the next cascade involves two enzymes in the linear sequence, there are four reaction steps to transform a methyl group to an amide (Scheme 15).^27^ The transformation of the methyl group to a carboxylic acid group involves actually three steps, namely C−H oxidation to the alcohol, oxidation to the aldehyde and finally to the acid, all catalyzed by a single enzyme (TtmP). The tandem oxidation followed by amidation of an ethyl group was reported for two‐step biosynthetic conversion of thiotetromycin to thiotetroamide C. When the overall transformation was performed in a sequential fashion 72 % yield (2.9 mg) for the first step (reaction a) and 93 % yield (2.7 mg) for the second step (reaction b) was achieved, resulting in 67 % overall yield. By running the cascade simultaneously (reaction c), 3.2 mg of final product was obtained corresponding to 80 % yield.

###### 3.1.2.2.3 Deamination or Hydrolysis Followed by Reduction

**Scheme 16:**
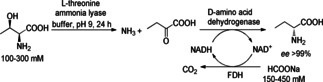
Cascade leading to D‐2‐aminobutyric acid from L‐threonine involving a L‐threonine ammonia lyase (L‐TAL), D‐amino acid dehydrogenase (D‐AADH), and formate dehydrogenase (FDH).

For the synthesis of D‐2‐aminobutyric acid with high optical purity, a simultaneous two step cascade with two enzymes in the linear sequence was developed (Scheme 16).^28^ Starting from threonine, the L‐threonine ammonia lyase from *Escherichia coli* (L‐TAL, *Ec*TAL) transformed the substrate to ammonia and the corresponding α‐keto acid. The latter was reductively aminated by the D‐amino acid dehydrogenase from *Symbiobacterium thermophilum* (D‐AADH‐*MSt*DAPDH). Without any addition of external ammonia, D‐2‐aminobutyric acid was obtained with >90 % yield, which demonstrates that the ammonia produced in the first reaction catalyzed by *Ec*TAL, can be used as the amino source in the reductive amination reaction and making the cascade more atomic economic. When the cell‐free extract or purified enzyme was used, the yield was >95 %, whereas with whole cells as biocatalyst the yield was less than 5 %. This may be due to a limited exchange of compounds through the cell membrane. It is worth to mention that with cell‐free extract, slightly lower *ee* values for the product were observed compared to the reaction with purified enzyme indicating the presence of other interfering enzymes present in the expression host. Although the substrate inhibited the second enzyme, the overall reaction was completed within 24 h. Since the first step went to completion already within 4 h, the inhibiting substrate for step two got removed.

**Scheme 17:**
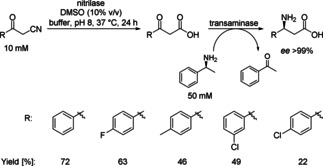
Two step simultaneous biotransformation leading to chiral (*S*)‐*β*‐amino acids from *β*‐keto nitriles hydrolysis by the nitrilase from *Bradyrhizobium japonicum* (NitBJ) and amination by the transaminase from *Polaromonas* sp.

The combination of a nitrilase with a reductive amination enabled the synthesis of enantiomerically pure (*S*)‐*β*‐amino acids (Scheme 17).^29^ *β*‐Keto nitriles were initially hydrolyzed to *β*‐keto acids using the nitrilase from *Bradyrhizobium japonicum* USDA 110 (NitBJ) and subsequently *β*‐keto acids were converted to *β*‐amino acids using transaminases. By using 3‐phenyl‐3‐oxopropanenitrile as the model substrate (10 mM), NitBJ (30 mg_DCW_/mL) and a transaminase from *Polaromonas* sp. (20 mg_DCW_/mL) the highest analytical yield (45 %) was obtained.

###### 3.1.2.2.4 Decarboxylation Followed by Oxidative C=C Cleavage

**Scheme 18:**
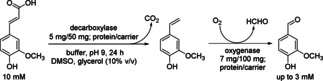
Coenzyme‐independent synthesis of vanillin from ferulic acid *via* 4‐vinylguaiacol by decarboxylase (ferulic acid decarboxylase Fdc from *Bacillus pumilus*) and oxygenase (4‐vinylguaiacol oxygenase Cso2 from *Caulobacter segnis*).

Two coenzyme‐independent enzymes, namely the decarboxylase Fdc (ferulic acid decarboxylase Fdc from *Bacillus pumilus*) and the oxygenase Cso2 (4‐vinylguaiacol oxygenase Cso2 from *Caulobacter segnis*), were immobilized on Sepabeads EC‐EA anion‐exchange carrier and used for the synthesis of vanillin *via* a simultaneous two‐step cascade starting from ferulic acid (Scheme 18).^30^ The immobilized enzymes could be recycled for four cycles leading each time to a similar amount of vanillin (3.1–3.5 mM). In subsequent reaction cycles, a decrease of the amount of produced vanillin and accumulation of the intermediate 4‐vinylguaiacol was observed. At the 1 mL scale, 2.5 mg vanillin was produced from ferulic acid by the immobilized Fdc and Cso2 catalysts after ten reaction cycles.

##### 3.1.2.3 Non‐redox Cascade: Hydrolysis and Amide Formation

**Scheme 19:**
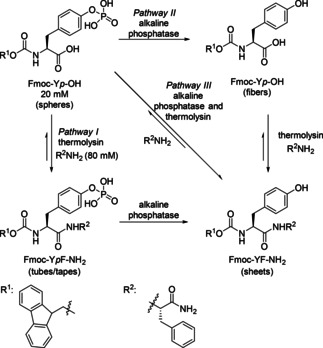
Schematic representation of sequential (*Pathways* I and II) and competing (*Pathway* III) biocatalytic pathways starting from precursors 9‐fluorenyl methyloxycarbonyl‐tyrosine (Fmoc‐Y*p*‐OH) and phenylalanine amide (R^2^NH_2_).

While in the previous chapters about two enzymes in the linear sequence, always one redox step was involved, the following sequence is void of a redox step. Starting from Fmoc‐tyrosine phosphate (Fmoc‐Y*p*‐OH, 20 mM) and phenylalanine amide (80 mM) in the presence of alkaline phosphatase (ALP) and thermolysin, four different self‐assembling molecules were formed (Scheme 19).^31^ ALP dephosphorylated tyrosine phosphate, while thermolysin catalyzed reversible amide hydrolysis and condensation. Three pathways were investigated involving either, sequential enzyme addition (*Pathways I* and *II*) or one‐pot system where both enzymes were added simultaneously but at varied ratios (*Pathway III*). When both enzymes were used in 1 : 1 (U/U) ratio, the system was first dominated by thermolysin activity, leading to 85 % conversion to Fmoc‐Y*p*F‐NH_2_ in 5 minutes (*Pathway III* was similar to *Pathway II*) and subsequent alkaline phosphatase‐catalyzed dephosphorylation to 56 % of Fmoc‐YF‐NH_2_ after 1 week. At the lowest thermolysin concentration (1 : 100), *Pathway III* resembled *Pathway I*. The initial stage of the cascade was controlled by ALP, as could be seen by the formation of Fmoc‐Y‐OH, which was followed by thermolysin catalyzed condensation, leading to the final composition of supramolecular products after 1 week: Fmoc‐Y‐OH (65%), Fmoc‐Y*p*F‐NH_2_ (11%) and Fmoc‐YF‐NH_2_ (24%).

#### 3.1.3 Three Catalysts in the Linear Sequence

**Scheme 20:**
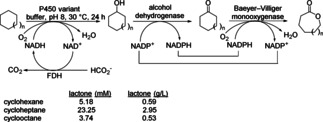
One‐pot conversion of cycloalkanes to the corresponding lactones using P450 BM3M NADH, *TeS*ADH and CHMO M16.

Most of the cascades involving three enzymes are based on cascades previously reported with two enzymes. For instance the two catalyst cascade described in Scheme 5 was extended by a cytochrome P450 monooxygenase (CYP450) for initial oxyfunctionalization of cycloalkane to cyclohexanol (Scheme 20).^32^ The co‐factor dependence of the hydroxylation step was uncoupled from step two and three by employing formate dehydrogenase (FDH) for NADH regeneration instead of glucose dehydrogenase, since the latter is not cofactor specific, thus it accepted both NAD^+^ and NADP^+^. Starting from cycloheptane a final product concentration of nearly 3 g/L 2‐oxocanone was obtained within 12 h which corresponds to a total turnover number (TTN) of 4185 with respect to the CYP450.

**Scheme 21:**
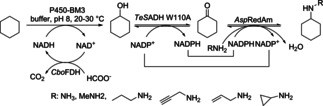
Biocatalytic cascade for the conversion of cycloalkanes to secondary amines employing a cytochrome P450 mono‐oxygenase for the first oxidation step followed by amination using an ADH in combination with a RedAm.

A related cascade was reported combining hydroxylation of cycloalkane in the first step with an alcohol dehydrogenase and a reductive aminase (RedAm) for the synthesis of secondary amines (Scheme 21).^16^ Amine product concentrations of up to 19.6 mM were obtained. The preparative scale amination of cyclohexane was also demonstrated with a space−time yield of 2 g/L.d. Different amine donors (10‐250 mM) including ammonia, methylamine, propylamine, allylamine, propargylamine and cyclopropylamine were tested. The cascade was run both in a simultaneous as well as two step fashions by using cell free extracts or purified enzymes. By using 100 mM of propargylamine the highest concentration of cyclohexylamine (19.6 mM) was obtained when running the cascade in a two‐step fashion.^16^

**Scheme 22:**
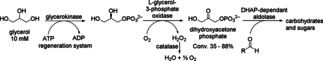
Four step, three‐enzyme cascade in the linear sequence incorporating *in situ* formation of dihydroxyacetone phosphate (DHAP) from glycerol *via* phosphorylation and oxidation and followed by aldol coupling and phosphate hydrolysis.

Enzymatic aldol reactions catalyzed by dihydroxyacetone phosphate‐dependent aldolases combine C−C bond formation with the generation of two new stereocenters. However, providing the required dihydroxyacetone phosphate (DHAP) for DHAP‐dependent aldolases in biocatalytic processes remains challenging due to its instability. An example cascade for DHAP production comprised a four‐enzyme reaction involving glycerol kinase, acetate kinase, glycerophosphate oxidase and catalase. This sequence was coupled with the DHAP‐dependent fructose‐1,6‐biphosphate aldolase to demonstrate the production of several rare chiral sugars (Scheme 22).^33^

**Scheme 23:**
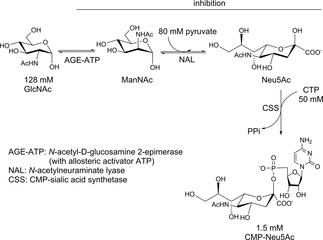
Three‐step synthesis of CMP‐*N*‐acetylneuraminic acid (CMP‐Neu5Ac) from *N*‐acetylglucosamine (GlcNAc), pyruvate, and cytidine triphosphate (CTP), whereby inhibition is circumvented by separation of AGE by compartmentalization.

The application of cascades may be limited in case one compound is toxic for an enzyme of the sequence or due to inhibitory effects. To overcome those inconveniences, reaction compartmentalization might be applied. One option is the implementation of enzymatic cascades in polymer vesicles formed from block copolymers that self‐assemble in aqueous solution, so‐called polymersomes. By equipping poly(methyloxazoline)_15_‐poly(dimethylsiloxane)_68_ poly(methyloxazoline)_15_ polymersomes with highly selective channel protein OmpF G119D, the three‐step synthesis of CMP−*N*‐acetylneuraminic acid (CMPNeu5Ac) in compartmentalized reaction spaces was investigated (Scheme 23).^34^ Mutant K160I of the *N*‐acyl‐D‐glucosamine 2‐epimerase (AGE) from *Anabaena variabilis*, which was separated from the incompatible component cytidine triphosphate (CTP) by encapsulation in polymersomes, was employed for epimerization of *N*‐acetyl‐D‐glucosamine (GlcNAc) to *N*‐acetyl‐D‐mannosamine (ManNAc). Channel proteins enabled the diffusion of GlcNAc and ManNAc inside and outside of the polymersomes, so only the first reaction step took place inside these nano‐reactors. Neu5Ac was formed by an *N*‐acetylneuraminate lyase from *E. coli* K12 (NAL)‐catalyzed aldol condensation of ManNAc and pyruvate. Subsequently, Neu5Ac was activated by sialic acid synthetase (CSS) derived from *Neisseria meningitidis* with CTP to form CMP‐Neu5Ac. NAL and CSS were immobilized on the outer surface using hydrophobic peptide anchors. The functionality of the nano‐reactors was proven by preparing 1.5 mM CMP‐Neu5Ac from 128 mM GlcNAc, 80 mM pyruvate and 50 mM CTP within 93 h.

With the application of enzyme membrane reactors (EMRs), a similar concept was reported for the two‐step synthesis of Neu5Ac.^35^ For continuous operation mode, the reactor was filled with 15.5 mL potassium phosphate buffer (100 mM, pH 7.5, T=30 °C) containing the substrates GlcNAc (50 mM) and pyruvate (100 mM), as well as the allosteric activator ATP (1 mM) without consuming it. The reaction was started by the addition of enzymes. After a batch phase of 3 h, the continuous operation was started for a long‐term operation (>85 h) leading to 35 % conversion, 28 % yield and a space‐time yield of 104 mg Neu5Ac 1/L.h. Since AGE was inhibited by pyruvate and Neu5Ac, cascades of EMRs employing both enzymes in each reactor were implemented to reduce the influence of a product inhibition. Neu5Ac was obtained as the final product at lower conversion (33 %) and yield (25 %), but better space‐time yield (199 mg Neu5Ac 1/L.h).^35^

#### 3.1.4 Five Catalysts in the Linear Sequence

**Scheme 24:**
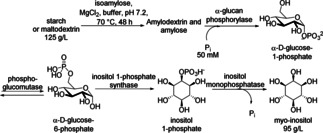
*In vitro* synthetic biology platform for the synthesis of inositol.

The conversion of starch to value‐added myo‐inositol was reported through an *in vitro* non‐fermentative five‐enzyme pathway (Scheme 24).^36^ This co‐factor independent pathway comprised five enzymes that operate without ATP or NAD^+^ supplementation. The cascade was initiated by a hyperthermophilic isoamylase (IA) from an archaeon *Sulfolobus tokodaii*, used to debranch α‐1,6‐glucosidic linkages, followed by α‐glucan phosphorylase from *Thermotoga maritima*, producing glucose 1‐phosphate. In the next step, phosphoglucomutase from *Thermococcus kodakarensis* transformed G1P to glucose 6‐phosphate. Then, inositol 1‐phosphate synthase from *Archaeoglobus fulgidus* isomerized G6P to inositol 1‐phosphate (I1P), which was subsequently dephosphorylated by inositol monophosphatase from *T. maritima* to inositol and phosphate. Finally, 125 g/L maltodextrin was efficiently converted to inositol in 20,000‐L bioreactor at 70 °C, leading to inositol concentration more than 95 g/L after 48 h reaction time.

An alternative pathway leading to myo‐inositol and fructose was published by the same group, based on four‐enzyme mediated conversion of 50 mM sucrose.^37^ To enable the use of less thermostable sucrose phosphorylase immobilized on cellulose‐containing magnetic nanoparticles, a switch controlled magnetic field was established. After two cycles (108 h), the final inositol concentration and yield were 48 mM and 98 %, respectively and in the third cycle, the final inositol yield reached nearly 100 %.

### 3.2 Sequential Mode

The most common reasons for performing a cascade in sequential mode are (i) different optimal reaction conditions of biocatalysts from different sources, (ii) inhibition of the enzymes by reagents/products from another step, (iii) possible undesired reactions due to insufficient substrate specificity. Biotransformations separated in time are termed “stages”, while one stage may comprise one or more reaction steps. Usually, to overcome reactions incompatibility, some actions have to be taken, like reagents addition at a later point of time, pH adjustment, unreacted substrates removal *etc*.^38^

#### 3.2.1 Two Catalysts in the Linear Sequence

**Scheme 25:**
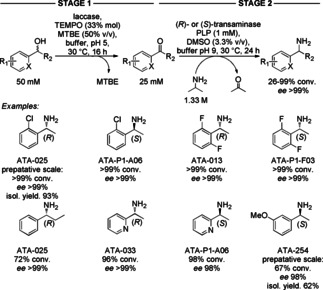
Sequential two‐enzymatic transformation of racemic alcohols into optically active amines using a laccase and a transaminase.

For instance, in the first example the reaction conditions were not compatible. One reason is different pH optima, another reason is the reagent for the second step (2‐propyamine), which would be oxidized by the enzyme of the first step. Therefore, this asymmetric amination of secondary alcohols was based on a sequential process involving the use of a laccase from *Trametes versicolor*/TEMPO catalytic system for the non‐stereoselective oxidation of alcohols to the corresponding ketones, followed by stereoselective amination to the corresponding amines by transaminases (Scheme 25).^39^ Under optimized reaction conditions, racemic substituted (hetero)aromatic *sec*‐alcohols were converted into amines with good to excellent optical purity (90–99 % *ee*) and conversions ranging from 67 to 99 %. To overcome the incompatibility of the two reaction steps, the amine donor was added once the oxidation step was completed and the pH was adjusted through the addition of phosphate buffer (200 mM, pH 9.0) to the reaction medium.

**Scheme 26:**
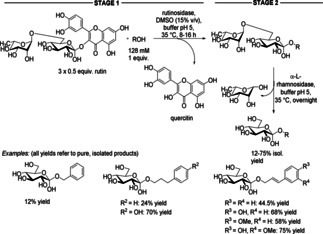
Two‐enzyme glucosylation of arylalkyl alcohols, exploiting rutin as a glycosyl donor.

To overcome the incompatibility of the two steps in the following cascade, the enzyme of the first step was thermally inactivated after completion of its job. In this one‐pot, two‐enzyme protocol, the glucosylation of arylalkyl alcohols produced two types of glycosylated products, namely rutinosides and *β*‐glucopyranosides (Scheme 26).^40^ The cascade was initiated by a transglycosylation catalyzed by a rutinosidase from *A. niger* using the cheap and commercially available natural flavonoid rutin as glycosyl donor. This step was followed by selective cleavage of the α(6→1) rhamnosyl unit of rutin catalyzed by a α‐l‐rhamnosidase from *A. terreus* to yield isoquercitrin. Rutinosidase was thermally inactivated prior to adding the rhamnosidase. The advantage of the biocatalytic approach is that it reduces the number of steps from 7‐steps in the chemical process to a 2‐step procedure. The products were isolated in up to 75% yield without silica gel chromatography.

#### 3.2.2 Three Catalysts in the Linear Sequence

**Scheme 27:**
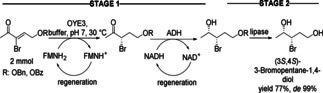
Three‐enzyme, one‐pot enzymatic cascade leading to bromohydrins.

Three catalysts in a sequential mode allow various combinations. For instance, to combine two catalysts as a simultaneous cascade at the first stage followed by an incompatible hydrolytic stage is exemplified in the first cascade. The incompatibility results from the general hydrolytic activity of the lipase for esters. The diastereoisomers of bromohydrins were obtained from an α‐bromo‐α,β‐unsaturated ketone as precursor (Scheme 27).^41^ The sequence was initiated by a C=C double bond reduction catalyzed by recombinant ene‐reductase belonging to the Old Yellow Enzymes family (OYE3). Subsequently, the carbonyl group was reduced by an alcohol dehydrogenase (ADH). The ene‐reductase and the ADH could be used in a simultaneous fashion, since the ADH was chemoselective for saturated ketones. Thus, the unsaturated ketone was not transformed avoiding formation of the allylic alcohol side‐product. With the optimized experimental conditions, the simultaneous cascade afforded *syn*‐ and *anti‐*configured bromohydrins in good yields (over 80 %) and excellent *de* (99 %). Enzyme catalyzed cleavage of the protecting benzoate ester group was initiated by adding the lipase from *Candida rugosa* to the reaction mixture at the end of the first stage. With the sequential process, an overall yield of 77 % for the (3*S*,4*S*)‐3‐bromopentane‐1,4‐diol without column purification was achieved.

**Scheme 28:**
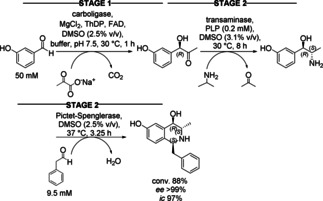
Enzymatic three‐step cascade leading to isoquinoline derivatives.

Three catalysts allow to perform a reaction sequence also in three stages as shown in the next example where the reagent of the second step would interfere with the first step and the reagent of the third step with the second step. After each of the first two steps the catalyst was removed by ultrafiltration, which is actually in the very strict sense not a cascade anymore. The biosynthesis of 1,3,4‐trisubstituted tetrahydroisoquinolines (THIQs) with three stereocenters was achieved by combining three types of enzymatic reactions (Scheme 28).^42^ The (*R*)‐selective, ThDP‐dependent acetohydroxy acid synthase I from *Escherichia coli* (*Ec*AHAS−I) catalyzed the C−C bond formation step between 3‐hydroxybenzaldehyde and pyruvate. The (*S*)‐selective transaminase from *Chromobacterium violaceum* (*Cv*2025) converted the hydroxyketone intermediate into the corresponding hydroxyamine using isopropylamine as the amine donor. Subsequently, the Pictet−Spenglerase variant derived from *Thalictrum flavum* (TfNCS−A79I) and phenylacetaldehyde was added to the reaction mixture leading to the tetrahydroisoquinoline (THIQ) scaffold. The overall cascade conversion of 3‐hydroxybenzaldehyde into (1*S*,3*S*,4*R*)‐THIQ was 88 % with excellent optical purity >99 % *ee*. In a similar sequential process, a (*R*)‐selective pyruvate decarboxylase and (*S*)‐selective transaminase were used for the stereoselective biosynthesis of l‐norephedrine.^43^

### 3.3 Multi‐enzyme Cascades in Flow

Over the last decade, flow systems were developed as an advanced modular concept for the multistep synthesis of organic compounds. The high level of safety, the possibility for automation, the high degree of process control, and profound process reliability have made continuous processes very appealing. Furthermore, continuous‐flow catalysis provides an additional benefit because it closes the gap between bench chemistry and chemical engineering by mimicking large‐scale production on the laboratory scale.^44^

**Scheme 29:**
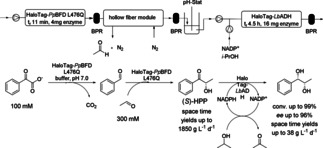
Enzymatic two‐step cascade for the continuous production of (1*S*,2*S*)‐1‐phenylpropane‐1,2‐diol (BPR: back‐pressure regulator; *Lb*ADH: *Lactobacillus brevis* alcohol dehydrogenase; *Pp*BFD L476Q: benzoylformate decarboxylase from *Pseudomonas putida* variant L476Q).

For performing biocatalytic reactions in flow, the enzymes need to be retained in the reaction vessel, which can either be achieved by using membranes and/or by immobilization of the enzyme on a carrier. For covalent immobilization for instance the HaloTag™ system was employed to bind enzymes directly from crude cell extracts in a packed‐bed reactor.^45^ Immobilization was performed on commercially available HaloLink™ Resin (Promega), which consists of a sepharose carrier exposing chloroalkane residues. Those residues were specifically recognized by the HaloTag™ and a covalent ester bond was established for immobilization of fusion enzymes. DNA encoding for the HaloTag™ was genetically fused to the N‐terminus of *Lactobacillus brevis* alcohol dehydrogenase (*Lb*ADH) or a variant of the thiamine diphosphate (ThDP)‐dependent benzoylformate decarboxylase from *Pseudomonas putida* (*Pp*BFD L476Q). The resulting tagged enzymes were produced in *E. coli* cells and immobilized directly in flow from *E. coli* crude cell extracts. Subsequently, a continuous enzymatic cascade was run leading to a chiral vicinal diol (Scheme 29). In the first step, 100 mM benzoyl formate was decarboxylated *in situ* to benzaldehyde by immobilized HaloTag‐*Pp*BFD L476Q. The resulting benzaldehyde was then converted by the same enzyme to (*S*)‐2‐hydroxy‐1‐phenylpropane‐1‐one ((*S*)‐HPP) by carboligation in the presence of 300 mM acetaldehyde. For optimal reduction activity in the last step, residual acetaldehyde was removed by membrane‐supported stripping with reversed nitrogen flow using a hollow fiber module and the pH was automatically adjusted to pH 7.0 with a pH‐Stat. (*S*)‐HPP was reduced to (1*S*,2*S*)‐1‐phenylpropane‐1,2‐diol by *Lb*ADH/NADPH, which was recycled using 10 % v/v of 2‐propanol as hydride source. Overall, a space−time yield of 1850 g/L.d with an operational stability of 3 days and an enzyme specific productivity of 488 g_product_/g_enzyme_ were achieved for the first cascade step. The second step with a space−time yield of 38 g/L.d, an operational stability higher than 14 days and an enzyme specific productivity higher than 48 g_product_/g_enzyme_ resulted in the efficient stereoselective production of (*S,S*)‐PPD, (*ee*/*de*) up to 96 %.^45^

**Scheme 30:**
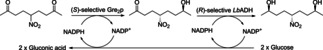
Sequential enzymatic reduction of the prochiral Cs‐symmetrical 5‐nitrononane‐2,8‐dione (NDK‐1) enables the stereoselective synthesis of the stereoisomeric hydroxyketones and diols: three‐enzyme, two‐step reaction for the reduction of NDK‐1 to the *meso* compound (diol).

In another immobilization approach, self‐immobilizing fusion enzymes were used for the modular configuration of microfluidic packed‐bed reactors. The three enzymes, the (*R*)‐selective alcohol dehydrogenase (*Lb*ADH), the (*S*)‐selective methylglyoxal reductase (Gre_2_p) and the NADP(H) regeneration enzyme, glucose 1‐dehydrogenase (GDH) were genetically fused with streptavidin binding peptide and halo‐based tags, to enable their specific and directional immobilization on magnetic microbeads coated with complementary receptors (Scheme 30).^46^ This enabled the (*R*)‐ or (*S*)‐selective reduction of the prochiral C_S_‐symmetrical substrate, 5‐nitrononane‐2,8‐dione (NDK) by using enzyme‐modified beads (Gre_2_p‐SBP@MB‐STV or *Lb*ADH‐SBP@MB‐STV) loaded in four‐channel microfluidic chips. The system was operating for up to 14 days. The overall product distribution could be controlled by fine‐tuning of compartment size and loading. For instance, by using 4 : 1 ratio of Gre_2_p/*Lb*ADH, the system was pushed to produce selectively a single meso diol with nearly quantitative conversion (>95 %) and excellent stereoselectivity (*dr* >99 : 1).

**Scheme 31:**
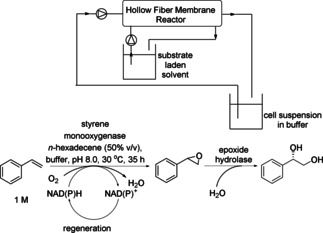
Hollow fiber membrane bioreactor (HFMB)‐based biphasic system for cascade biotransformations.

Styrene exhibits toxicity for certain enzymes. This is also a challenge for the enantioselective *trans*‐dihydroxylation of styrene with recombinant cells of *E. coli* (SSP1) co‐expressing styrene monooxygenase (SMO) and *Sphingomonas* epoxide hydrolase (*Sp*EH) for production of (*S*)‐1‐phenyl‐1,2‐ethanediol (Scheme 31).^47^ To solve this problem, the aqueous and organic phases were separated by a membrane. By using a hollow fiber membrane bioreactor (HFMB)‐based biphasic system, the majority of the substrate was present in *n*‐hexadecane in the shell side to avoid toxicity, while the majority of the product remained in aqueous cell suspension phase of the lumen side providing high productivity and easy product recovery. The established HFMB‐based biphasic system enhanced the product concentration to 143 mM, being 16‐fold higher than the aqueous system (8.75 mM) and 1.6‐fold higher than the traditional dispersive partitioning biphasic system (TDPB, 89.1 mM). Furthermore, the combination of the biphasic system with HFMB prevented the foaming and emulsification, thus facilitating the downstream purification.^47^

**Scheme 32:**
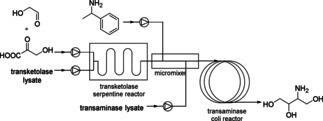
Enzymatic synthesis of chiral amino‐alcohols by coupling transketolase and transaminase‐catalyzed reactions in a continuous‐flow microreactor system.

To circumvent incompatibilities of reagents within different steps, flow concepts are also suitable to achieve compartmentalization. In the following example, the amination step (second step) is not compatible with the substrates of the first step, since they could be aminated as well. The target products of the following cascade are chiral amino‐alcohols which represent key industrial synthons for the production of complex molecules and optically pure pharmaceuticals. Especially, (2*S*,3*R*)‐2‐amino‐1,3,4‐butanetriol (ABT) represents a highly valuable building block for the synthesis of statins^48^ and is an intermediate in the synthesis of detoxinine.^49^ By using a continuous‐flow microreactor‐based approach, full conversion into ABT was reported (Scheme 32).^50^ The two‐step cascade in flow was performed by coupling a transketolase (TK)‐catalyzed asymmetric carbon‐carbon bond formation with a transaminase (TAm)‐catalyzed conversion of the keto‐group into a chiral amino group. The compartmentalization allowed under optimized reaction conditions to complete the transketolase‐catalyzed step within 10 min with a volumetric activity of 3.25 U/ml and the transaminase‐catalyzed step with a volumetric activity of 10.8 U/ml within 2 h.^50^

## 4 Artificial *in vivo* Cascades

Nature provides a highly efficient but complex and often sensitive reactor system in the form of living cells. In these natural bioreactors, multistep reactions are catalyzed by numerous enzymes in an aqueous environment. However, this approach is mostly limited to already‐existing metabolic pathways and does not necessarily deliver the high structural diversity of compounds utilized by the chemical industry. To produce pharmaceutically and industrially relevant synthetic products, synthetic biology methodologies might be applied.^51^ In this approach, the construction and optimization of the system is achieved by genetic manipulation and the cascade requires only starting material and the whole cell catalyst with cofactors provided internally by the metabolism. In contrast to classical metabolic engineering, where only the cell‘s metabolism is engineered towards a certain metabolite, synthetic biology aims to introduce cascades that have no biological origin.^52^ Despite the *in vivo* system being more complicated than *in vitro,* the overall benefit of having the single catalyst that provides all necessary cofactors by the metabolism makes it very attractive. However, *de novo* designed synthetic cascades can potentially raise problems in whole cells, such as metabolic degradation, generation of reactive intermediates, membrane transport and activity control of individual biocatalysts.^53^

### 4.1 Two Catalysts in the Linear Sequence

**Scheme 33:**
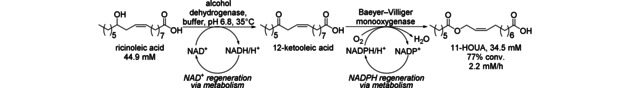
Biotransformation of ricinoleic acid to (E)‐11‐(heptanoyloxy) undec‐9‐enoic acid (11‐HOUA) using resting *Escherichia coli* BL21(DE3) cells co‐expressing Adhp and BVMO.

Improvement in the conversion of ricinoleic acid into (*E*)‐11‐(heptanoyloxy) undec‐9‐enoic acid (11‐HOUA) was recently published.^54^ While previously the biotransformation was carried out without addition of energy sources leading to the deactivation of the resting cells because of limited amounts of intracellular cofactors, a process was designed using continuous feeding of an energy source such as glucose and glycerol. Fed‐batch cultivation of recombinant *E. coli* overexpressing an alcohol dehydrogenase from *Micrococcus luteus* (Adhp) and a Baeyer‐Villiger monooxygenase (BVMO) from *Pseudomonas putida* KT2440 was carried out to achieve high‐density of the single cell biocatalyst. As shown in Scheme 33, ricinoleic acid was oxidized to 12‐ketooleic acid by Adhp. 12‐Ketooleic acid was further oxidized into 11‐HOUA by BVMO using O_2_ and NADPH cofactor. High‐cell preparation with glucose feeding at the cell growth stage and intermittent glycerol supplementation at the biotransformation stage resulted in 34.5 mM of 11‐HOUA (equivalent to 10.8 g/L) from 44.9 mM ricinoleic acid with 77 % conversion and 2.2 mM/h of productivity, which corresponded to 1.7‐folds higher values than those in the batch‐type biotransformation.^54^

### 4.2 Three Catalysts in the Linear Sequence

**Scheme 34:**
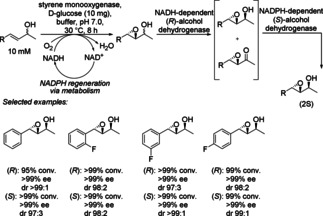
The synthesis of optically pure *sec*‐epoxy alcohols from racemic allylic alcohols using a single whole‐cell biocatalyst of recombinant *E. coli* co‐expressing three oxidoreductases.

Compared to the classic Sharpless kinetic resolution, the synthesis of optically pure secondary epoxy alcohols from racemic allylic alcohols using a single whole‐cell biocatalyst does not require a metal catalyst or organic solvent (Scheme 34). The cascade was enabled by recombinant *E. coli* co‐expressing three oxidoreductases, a styrene monooxygenase catalyzing the formation of the chiral epoxy group, and two alcohol dehydrogenases catalyzing the epimerization of the hydroxy group and subsequent oxidation reaction. Two stereo‐complementary alcohol dehydrogenases may be applied in order to obtain either the (*R*)‐ or the (*S*)‐configured product. To be able to control which ADH is performing the enantioselective oxidation and which the stereoselective reduction, the two ADHs dependent reactions have to rely on complementary cofactor, thus one on NAD^+^ the other on NADPH. Excellent enantio‐ (up to >99 % *ee*) and diastereoselectivities (dr >99 : 1) were achieved for 12 substrates.^55^

**Scheme 35:**
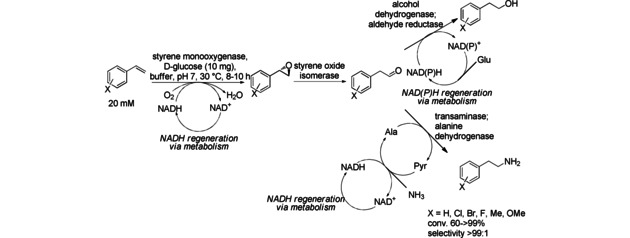
Epoxidation‐isomerization‐amination and epoxidation‐isomerization‐reduction cascade.

Two important anti‐Markovnikov reactions are hydroamination and hydration to produce terminal amines and alcohols, respectively (see also Scheme 8). To address this challenge, two biocatalytic concepts involving enzyme cascades were examined: epoxidation−isomerization−amination for formal hydroamination and epoxidation−isomerization−reduction for overall formal hydration (Scheme 35). An *E. coli* strain co‐expressing styrene monooxygenase (SMO), styrene oxide isomerase (SOI), transaminase (*Cv*TA), and alanine dehydrogenase (AlaDH) was applied for the hydroamination of styrene derivatives, leading to the corresponding terminal amines with high conversion (from 45 to >99 %) and exclusive anti‐Markovnikov selectivity (>99 : 1). Another *E. coli* strain co‐expressing SMO, SOI, and phenylacetaldehyde reductase (PAR) catalyzed the hydration of the same styrene derivatives to their corresponding terminal alcohols with high conversion (from 60 to >99 %) and excellent anti‐Markovnikov selectivity (>99 : 1).^56^

**Scheme 36:**
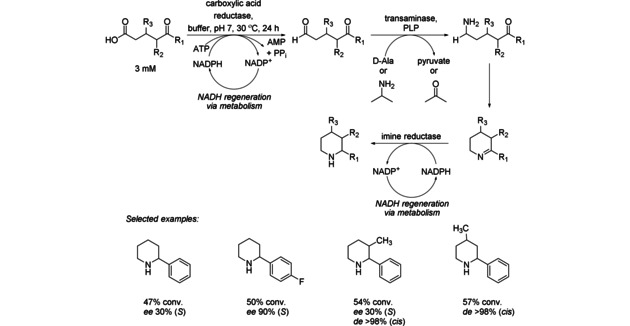
Three‐enzyme cascade for the diastereoselective and/or enantioselective conversion of linear keto acids into cyclic amine products.

Three enzymes in the linear sequence are also required for the diastereoselective and/or enantioselective conversion of linear keto acids into cyclic amine products (Scheme 36). The pathway starts with carboxylic acid reduction to the corresponding keto aldehyde catalyzed by carboxylic acid reductase (CAR) that triggers a transaminase‐catalyzed transamination, spontaneous imine formation, and subsequent imine reduction by imine reductase (IRED). A variety of parameters concerning both protein expression and reaction conditions were investigated. Finally, the best results were obtained when duplication gene strategy was used. The addition of a duplicate (*S*)‐IRED gene into an original construct significantly improved the conversion of amines, circumvented the accumulation of imine intermediate and drove the reaction toward amine production. The use of a single whole‐cell system provided access to piperidines with high conversion (up to 93 %) and enantiomeric excess (up to 93 %).^57^

**Scheme 37:**
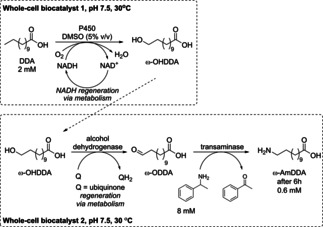
Schematic diagram of enzymatic synthesis of ω‐hydroxy dodecanoic acid (ω‐OHDDA) from dodecanoic acid (DDA), and ω‐amino dodecanoic acid (ω‐AmDDA) from ω‐hydroxy dodecanoic acids (ω‐OHDDA).

Another strategy providing an access to industrially important monomer for Nylon 12^58^ ω‐amino dodecanoic acid (ω‐AmDDA) was based on the combination of two different types of cells (Scheme 37). ω‐AmDDA was obtained from dodecanoic acid *via* hydroxylation to give ω‐hydroxy dodecanoic acid using whole‐cells expressing P450 (CYP153A) and CamAB. Then, the produced alcohol served as substrate for the second whole‐cell catalyst co‐expressing an alcohol dehydrogenase (AlkJ) and a transaminase. Within 6 h reaction time a 0.6 mM solution of ω‐AmDDA was obtained.^59^

## 5 Hybrid *in vitro* and *in vivo* Cascades

As an extension to the previous suggested classification,1a recent work has demonstrated that cascades can be developed that combine both *in vitro* biotransformations and *in vivo* cascades within the same system. This combination was employed for the preparation of 1,9‐nonanedionic acid. In a previous study the hydrolysis of plant oils was reported using the *Thermomyces lanuginosus* lipase (TLL) and subsequent C9−C10 double‐bond cleavage in unsaturated fatty acids. The cascade reaction encompassed a fatty acid double‐bond hydratase from *Stenotrophomonas maltophilia*, an alcohol dehydrogenase from *Micrococcus luteus* (SADH), and a Baeyer‐Villiger monooxygenase (BVMO) from *Pseudomonas putida* KT2440 expressed in *E. coli*.^60^

**Scheme 38:**
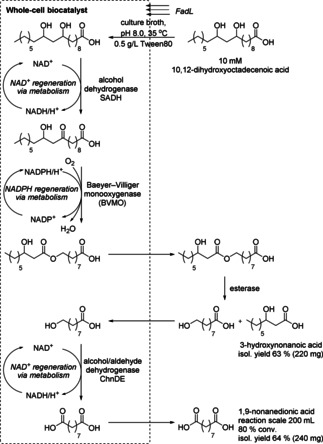
Combination of *in vitro* biotransformation (ester hydrolysis) and an *in vivo* cascade (*FadL*: fatty acid transporter; ChnDE: alcohol dehydrogenase from Actinobacter; SADH: alcohol dehydrogenase from *Micrococcus luteus*).

This concept was further extended by employing the alcohol dehydrogenase (ChnDE) from *Actinobacter* and the esterase from *P. fluorescens* in addition to the SADH and BVMO (Scheme 38). In more detail, the biotransformation was started by addition of 10 mM 10,12‐dihydroxyoctadecenoic acid and the esterase into the culture medium of the recombinant *E. coli* co‐expressing SADH, BVMO, ChDE and fatty acid transporter *FadL* in the outer membrane. The substrate was transformed into 10‐oxo‐12‐hydroxyoctadecanoic acid with high regioselectivity by SADH, followed by BVMO‐catalyzed oxygenation into 9‐(12‐hydroxynonanoyloxy)‐nonanoic acid. The corresponding ester was excreted and subjected to hydrolysis by the extracellular esterase. Then the hydrolysis product entered back into the *E. coli* cells and was further oxidized to 1,9‐nonanedionic acid by ChnDE. Preparative‐scale reaction (200 mL) afforded 3‐hydroxynonanoic acid and 1,9‐nonanedionic acid with 80 % conversion and isolated yields of 63 % and 64 %, respectively after purification by column chromatography.^61^

## Outlook

In summary, the development of artificial biocatalytic cascades is a rapidly expanding area of research,^62^ including many *in vivo, in vitro* and also hybrid cascades for the production of a wide range of valuable chemical scaffolds.

A special challenge is the case when reactions steps are not compatible to each other, which might be due to (i) differing optimal reaction conditions of each step (pH, T…), (ii) inhibition of the enzymes by reagents/products from another step, (iii) undesired reactions due to insufficient substrate specificity. The incompatibility may be addressed by performing the cascades in a sequential fashion, or compartmentalization or reactions, or enzyme engineering to adapt reaction optima or substrate specificity. Various examples in the review show that biocatalytic cascades performed simultaneously enabled to increase the yield of the final product compared to cascades performed in a stepwise fashion.

Currently, the majority of *in vitro* cascades encompasses two reaction steps in the linear sequence. Nevertheless, examples in this review demonstrate, that the two step cascades are the base to be extended to more steps (see e. g. Scheme 20 and 21). Implementing cascades *in vivo* enables multistep synthesis with the advantage of providing all necessary redox cofactors in host cell, however dealing also with a significant more complex system being limited by the stability of the whole‐cell catalyst under the conditions used and facing additional challenges such as transport of compounds *via* the cell membrane. Nevertheless, several whole‐cell systems co‐expressing three or more recombinant proteins have been already demonstrated successfully.

Although cascades have many advantages, a number of them still suffer from low substrate loadings,^63^ which limits the scalability of these reactions. To implement artificial biocatalytic cascades in industrial processes more widely, it is necessary to optimize the selected systems with respect to productivity, process cost, catalyst stability and recyclability.1u,^64^

It is worth to note, that for a certain multi‐step transformations different cascades have been developed like for the formal hydration of alkenes (see Scheme 9 and 35). The shorter cascade (Scheme 9) was enabled due to recent developments of a new reaction.^18^ Nevertheless, comparing the various reviews on cascades, certain transformations pop up several times, like the amination of alcohols„ transformation of alcohol to esters or the transformation of a CH_2_ moiety to a carbonyl (mostly ketone). These three transformations (and others may line up) seem to be highly appealing reactions. Therefore, it would probably be worth to consider these as modules of general significance for synthesis and make catalysts preparations for these modules broadly available (like a commercial enzyme). This would also allow to apply no specialist the usage of biocatalytic cascades. Such a step would probably even further push the application of bio‐cascades.

## Conflict of interest

The authors declare no conflict of interest.

## Biographical Information

***Somayyeh Gnadomkar** was born in Tehran, Iran, in 1986. She received her PhD in organic chemistry from Shahid Beheshti University (SBU, Tehran) in 2016. After that she started her postdoctoral research on fatty acid cascades with Prof. Kurt Faber at the University of Graz for one year. Afterwards, she has been working with Prof. Wolfgang Kroutil at the Universty of Graz as an Lise‐Meitner postdoctoral fellow on oxidation‐amination cascades. Her current research interests include organic synthesis, application of enzymes in organic synthesis, biocatalysis, enzyme engieering, ezymatic and chemo‐enzymetic cascades*.

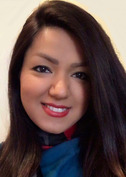

## Biographical Information

**Anna Żądło‐Dobrowolska** was born in 1986 in Warsaw, Poland. In 2016 she received her PhD at the Institute of Organic Chemistry Polish Academy of Sciences under the guidance of Prof. Ryszard Ostaszewski. Currently, she is an Lise‐Meitner postdoctoral fellow with Prof. Wolfgang Kroutil at the University of Graz. Her research focuses on the design, development and investigation of novel enzymatic and chemo‐enzymatic strategies for the synthesis and discovery of biologically active compounds. Current interests include the development of biocatalytic Friedel‐Crafts acylation.

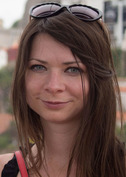

## Biographical Information

**Wolfgang Kroutil** is full professor and head of the Institute of Chemistry at the University of Graz. He received his undergraduate training in chemistry at the University of Technology in Graz (Austria) and conducted his PhD‐research in Exeter as well as in Graz (TU Graz). After two years in industry he became Ass. Prof. at the University of Graz, where he went up the ranks. His research deals with the development of biocatalytic methods for organic synthesis, especially redox reaction, C–C bond formation, cascades and asymmetric transformations